# Experiences of older adults who have lost a child: reflections on grief and for nursing

**DOI:** 10.1590/0034-7167-2025-0260

**Published:** 2026-07-06

**Authors:** Richard Augusto Thomann Beckert, Valéria Silvana Faganello Madureira, Ivonete Teresinha Schulter Buss Heidemann, Tiago Luan Labres de Freitas, Angélica Zanettini Konrad, Samille Isabel Palombit Ronsoni, Maiara Bordignon

**Affiliations:** I Universidade Federal de Santa Catarina. Florianópolis, Santa Catarina, Brazil; II Universidade Federal da Fronteira Sul. Chapecó, Santa Catarina, Brazil

**Keywords:** Bereavement, Aged, Health of the Elderly, Parent-Child Relations, Nursing., Aflicción, Anciano, Salud del Anciano, Relaciones Padres-Hijo, Enfermería.

## Abstract

**Objectives:**

to understand the experience of the grieving process in older adults who, at any stage of life, have lost a child.

**Methods:**

qualitative research involving eight older adults selected using the snowball sampling method. Semi-structured interviews were conducted and analyzed using the Discourse of the Collective Subject technique.

**Results:**

the themes included: Differences between grieving for a child and for other family members; Feelings triggered by death; Duration of feelings and grief; Feelings of grief that remain; and Ways of coping with a child’s loss.

**Final Considerations:**

although acceptance of loss is possible, longing and absence remain. The importance of faith and support networks in coping with grief was highlighted. Nursing can play an important supporting role in coping with grief. Understanding these experiences is fundamental to providing comprehensive care.

## RESUMEN

**Objetivos:**

comprender la experiencia del duelo en ancianos que, en cualquier etapa de la vida, han perdido un hijo.

**Métodos:**

investigación cualitativa con ocho ancianos seleccionadas mediante muestreo en cadena. Se realizaron entrevistas semiestructuradas y se analizaron utilizando la técnica del Discurso del Sujeto Colectivo.

**Resultados:**

los temas incluyeron: Diferencias entre el duelo por un hijo y por otros miembros de la familia; Sentimientos provocados por la muerte; Duración de los sentimientos y el duelo; Sentimientos de duelo que permanecen; y Formas de afrontar la pérdida de un hijo.

**Consideraciones Finales:**

si bien es posible aceptar la pérdida, la añoranza y la ausencia persisten. Se destacó la importancia de la fe y las redes de apoyo para afrontar el duelo. La enfermería puede desempeñar un papel fundamental de apoyo en este proceso. Comprender estas experiencias es esencial para brindar una atención integral.

## INTRODUCTION

The Brazilian population is aging due to a reduction in the birth rate and an increase in life expectancy, causing an inversion in the age pyramid, a phenomenon called “demographic transition”^(1)^. This change results in a growing demand for healthcare services and tools to improve older adults’ quality of life so that they can be active for longer^(1)^.

Aging begins before the age of 60, which defines individuals as older adults in Brazil^(2)^, and is characterized as a complex process of biopsychosocial changes that encompass social, economic, physical and psychological transformations^(3)^. Thus, senescence refers to natural aging, while senility is characterized by more pronounced losses due to acute or chronic health complications^(3)^.

Aging can be seen as a process of gains and losses, where success lies in an individual’s ability to adapt to the changes that accompany it. Losses during life can encompass various aspects, such as a shrinking social circle, reduced functional capacity, changing roles in society, and the death of acquaintances, friends, or family members^(4,5)^. Grief is a response to loss, marking the end of a particular way of relating between the bereaved individual and the deceased loved one, resulting in changes in the bereaved person’s life^(4,5)^.

Understanding grief can be difficult precisely because it is a subjective experience unique to a person going through it. However, psychiatrist Elisabeth Kübler-Ross, while working with terminally ill patients, developed a widely disseminated theory identifying five stages of the grieving process: denial; anger; bargaining; depression; and acceptance^(6)^.

According to these stages^(6)^, in the “denial” stage, an individual denies the loss as an initial and temporary defense; in the “anger” stage, the feeling is one of great revolt and indignation at the sense of injustice of the loss; the “bargaining” stage refers to the moment when a bereaved person’s feelings are mitigated and there is an attempt to negotiate the loss and reverse the situation; in the “depression” stage, a bereaved person realizes that there is no way to recover what has been lost and melancholy predominates; and in the “acceptance” stage, a bereaved person progressively resumes functionality in their life. Although not all bereaved people go through all the stages, which are cyclical and can alternate throughout the coping process^(7)^, it is evident that grief can bring diverse feelings and sensations, which have the potential to significantly influence the health of those who experience it.

Furthermore, human beings develop in a social environment, and the relationships between parents and children are generally characterized by affection and intimacy^(8)^. Thus, a child’s loss can be a painful process at any stage of life, with a more intense and prolonged grief compared to other losses^(8)^. As people age, many face a child’s loss and experience it in a unique way, which impacts their health and may require support from healthcare professionals^(9)^. Many illnesses begin in difficult situations that people go through, including the grieving process^(10)^. Professional support in these cases, characterized by listening and dialogue, is important to help people cope with these situations^(10)^.

Nursing professionals are on the front lines of healthcare services, placing them in direct contact with patients, families, and caregivers. Considering human care as the core of their profession, sensitivity to issues such as grief is essential for a broader understanding of the health-disease process, ensuring comprehensive and humane care^(11)^. In this context, the nurses’ role in caring for bereaved individuals involves a variety of skills, including soft care technologies such as listening, support, and communication, as well as the implementation of interventions and support programs for bereaved individuals, paying attention to their cultural and spiritual needs, among others^(12)^.

This context demonstrates the importance of developing, during nursing education and afterwards, when nursing professionals are working in healthcare services, knowledge and skills for welcoming and supporting bereaved individuals and families, ensuring attention to the particularities of each situation^(12)^. Given this, the following research question emerged: what was or is the experience of grief like for older adults who, at some point in their lives, lost a child?

To map the discussion of this topic at the national level, a brief search was conducted in the Coordination for the Improvement of Higher Education Personnel Thesis and Dissertation Database using the terms “grief”, “older adults”, and “children”. Nine results were found, ranging from 1997 to 2012, of which seven are academic master’s dissertations, one is a professional master’s thesis, and one is a doctoral dissertation. Modifying the search to the terms “grief”, “older adults”, and “nursing”, nine works developed between 2004 and 2009 were also found, including six master’s dissertations and three doctoral theses. Thus, given the scarcity of research, it is clear how much space there is still for academic discussion of grief in older adults who have lost a child, whether in health in general or in nursing.

## OBJECTIVES

To understand the grieving process experienced by older adults who, at any stage of life, have lost a child.

## METHODS

### Ethical aspects

The research was approved by the Research Ethics Committee of the authors’ affiliated university. It followed ethical guidelines for research with human subjects and ensured participant anonymity through the use of the letter “I”, for interviewee, followed by a number indicating the order of the interviews (I1; I2; I3; I4; I5; I6; I7; and I8).

### Study design

This is a qualitative study. This design consists of a series of different interpretive techniques with the aim of describing and decoding the components of a complex system of meanings^(13)^. This study report also considered the COnsolidated criteria for Reporting Qualitative research guide^(14)^.

### Study setting

The study included older adults from a municipality located in western Santa Catarina, Brazil, who had experienced grief following a child’s death.

### Data source

Eight older adults who had experienced grief over a child’s death participated in the study. The number of participants was determined by data saturation^(15)^, indicating the end of data collection when responses began to repeat and the meaning of the phenomenon/group studied was understood. Participants were selected using the snowball sampling method^(16)^, a sampling method based on successive references.

Initially, two older adults, known to the lead researcher from her theoretical and practical activities at a Basic Health Unit, were invited via a messaging app. Subsequently, these older adults indicated others who had experienced a child’s loss to be invited, continuing the invitation and inclusion process until reaching eight participants. Individuals over 60 years of age who had experienced a child’s loss during their lives were included in the study. Older adults who did not reside in the municipality studied were excluded. It is important to note that telephone contact was established with all participants before the interview to present the study, the interviewer, and to obtain their acceptance of participation, followed by the signing of an Informed Consent Form.

### Data collection and organization

Data collection was guided by a questionnaire developed for this study. Interviews were conducted by two of the authors in participants’ homes between July and October 2023. Participants received instructions regarding the recording of interviews and agreed to it. The interviewers were undergraduate nursing students, one male and one female, who worked under the direct supervision of a PhD nursing professor. Transcriptions were made immediately after each interview. It should be noted that, during data collection, nine older adults were contacted, but one of them refused to participate after two unsuccessful attempts to interview them.

### Data analysis

The data obtained were transcribed and analyzed according to the Discourse of the Collective Subject (DCS)^(17)^, a technique that analyzes testimonies and identifies key expressions, central ideas (CIs) and anchorages to understand the linguistic manifestations of thoughts about a CI. Initially, a thorough reading of each participant’s responses was carried out to identify key expressions, which represented the testimony’s essence. Subsequently, CIs were identified, which are synthetic expressions that convey the meaning of each discourse. Finally, the key expressions that shared the same CI were grouped to form the DCS^(17)^. From the analysis of interviews, nine themes, 25 CIs and 31 DCSs emerged, five of which, together with their respective CIs and DCSs, were explored in this study.

## RESULTS

Concerning the profile of the eight older adults, Chart 1 was created that presents age, sex, occupation, marital status, number of children, number of grandchildren, religion, and number of deceased children.

**Chart 1:** Participant demographic data

	Age	Sex	Occupation	Marital status	Number of children	Number of grandchildren	Religion	Deceased children
**E1**	66	F	Retired	Divorced	2	1	Catholic	1
**E2**	77	F	Retired	Widow	8	22	Catholic	1
**E3**	95	F	Retired	Widow	12	22	Catholic	3
**E4**	63	F	Retired	Divorced	2	0	Lutheran	2
**E5**	65	F	Retired	Widow and divorced	14	9	Catholic	6
**E6**	74	F	Babysitter	Divorced	6	5	Catholic	3
**E7**	62	M	Bricklayer	Widower	2	0	Catholic	1
**E8**	69	F	Retired	Widow	4	5	Catholic	1

Below are five themes that emerged from the analysis, along with their respective CIs and DCSs.

### (1) Differences between grieving for a child and for other family members

CI - There have been many losses, but losing a child is incomparable.

DCS - There were many losses, and they were heavy. I attended many funerals for relatives, but, for instance, with the death of parents, the grief is lighter because you think that they had to go, as I will have to go one day. When a husband or wife dies, the grief is more fleeting. But the experience of seeing your child come home, give you a hug, a kiss, ask for a blessing, that’s what makes the difference! Losing a child is incomparable! When you lose a child, it feels like a piece of you has died. The pain is much more in the heart. The suffering is much greater than in other griefs, because it feels like the heart is shattered, like there’s a wound.

CI - All losses are similar.

DCS - I think it’s all similar. For instance, the children share the husband’s blood. So, there’s no way to forget. We only forget after we die, right?

### (2) Feelings triggered by death

CI - At first, I was shocked and thought it was a joke.

DCS - At first, I was so shocked that I didn’t feel anything else and thought it was a joke. Then, despair set in; I couldn’t believe what was happening and almost fainted. I felt devastated. I felt a lot of pain in my heart for not being able to do anything and kept questioning why it had happened, without any answers.

CI - At that moment, my world ended, and only sadness remained.

DCS - My life stopped there, and I had no more feelings inside me. I didn’t think about what I had to do, I didn’t think about anything anymore, because my world closed down and only sadness remained, so much sadness. I feel like my world ended there. I was left without a foundation and I didn’t want to live anymore. It’s a very sad situation; my son was my pride and joy!

CI - Losing a child is even sadder when you have lived with them for a long time.

DCS - My dream was to have twins! I was blessed, but I didn’t get to live with them. So, if losing a newborn is already sad, imagine losing a child you knew so well!

### (3) Duration of feelings and grief

CI - Grief lasts a long time.

DCS - Grief lasts a long time. Before, I would despair! You can lose everything, but losing a child is especially difficult! I still have that tightness in my heart. I feel like I’m not the same person anymore, that I don’t have that joy I used to have.

CI - Grief and loss do not go away.

DCS 1 - Even today, I’m still grieving. It hasn’t passed and it won’t pass. I miss them, I long for them, and this feeling won’t go away. To this day, I wear black all the time and take medication for depression. My life is over. So, I don’t want to hurt anyone, but I want to die.
DCS 2 - I’ve learned to live with it, but his absence will be hard to overcome. I know life goes on, that just because my son passed away doesn’t mean I died too, right? I have other children, and that’s why I have to stay strong to get through this and support those who remain.

### (4) Feelings of grief that remain

CI - It is something you remember every day and miss.

*DCS - I remember it every day. There isn’t a moment when I forget about it! I try to overcome it as much as possible, but it’s always present in my life. There’s always that little pain, and it weighs on me. I feel a tired, heavy pain that only eases when I cry. It hurts me a lot. So, today the feeling is one of longing; I miss him so much. I miss his hugs and the moments we lived together.*

CI - I cannot accept the loss.

DCS 1 - I wish he were here with us. I can’t understand it! I can’t accept the loss. Sometimes I think I’ll die and I won’t be without him.
*DCS 2 - Sometimes I think he’s traveling and will come back, it seems like he’s coming home, but I know he’s not. I try to get over it, but saying he didn’t exist is impossible! I don’t know how to explain what’s happening. Today I don’t feel like doing anything, like working, but I’m not going to stay home doing nothing. I’ve lost my zest for life.*

### (5) Ways of coping with a child’s loss

CI - Faith, prayer, and positive thinking helped a lot.

DCS - First and foremost, a lot of faith in God! Prayer helps a lot, so I sought out the church a lot, and that helped me. I pray a lot, and one day I decided to do a novena to Our Lady of Caravaggio. That calmed my heart and I started sleeping well, crying less. So, it was faith and always thinking positively.

CI - Wearing a closed-off grief garment helped me.

DCS - In the old days, it was a strict grief period, wearing black clothes for six months to a year! Doing otherwise was against God. I think this helped, because I needed to get through that time, through that situation.

CI - Going to the cemetery comforts me.

DCS - There are days when I go to the cemetery. I stay there all day and I don’t feel like leaving because the grave I made for him calms my heart. Going there to clean the grave, talk to him and take care of him. I put a wreath of flowers, photos, and I keep visiting the cemetery to pray. That comforts me.

CI - I do many things to help myself.

*DCS 1 - I try to crochet, visit a friend to calm myself down and not have my thoughts only on that* [her son’s death]*. Besides that, I try to work, keep doing things and move on with life as best I can. I try, as much as possible, to be strong, because if I didn’t help myself and have strength, I wouldn’t be able to overcome it, right?*
DCS 2 - After losing my newborn son, I throw a small party for 12 children on Children’s Day. I give gifts and do my best to please them. I do this to try to fill that void that never fills.

**Figure 1:**
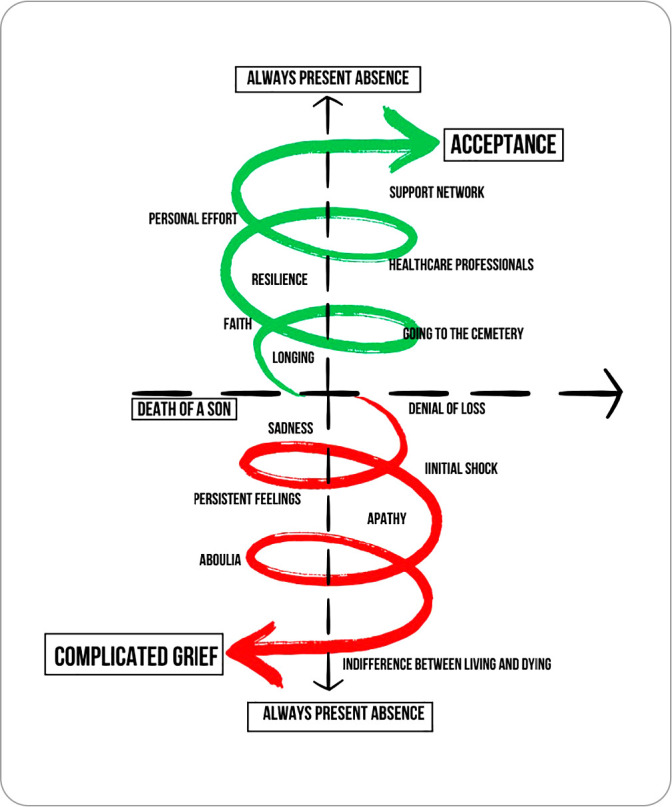
Graphic representation of the grief experienced after a child’s loss, Chapecó, Santa Catarina, Brazil

Sequentially, Figure 1 graphically represents the impacts of a child’s loss on older participants’ lives. A horizontal dotted line symbolizes the death of the child, while two spiral arrows represent the grieving process. The ascending spiral, colored green, represents a grieving process without complications, while the descending spiral, in red, represents a complicated grieving process. A vertical line cuts through the spirals, symbolizing the permanent sense of loss felt by older adults after a loss. The words on the spirals represent elements associated with the experience of grief, as described in the results.

## DISCUSSION

For someone over 60, a family member’ s loss and the experience of grief are generally familiar occurrences^(18)^. The impact that the loss will have on a bereaved person’s life is related to the bond and affection that existed between the loved one and the person who remains. The bond between parents and children is often strong in affection, such that its rupture causes great suffering and may require greater effort to adapt to the new reality and to process the grief^(9,19)^.

During the interviews, when asked about other experiences of significant loss in their lives, the older adults highlighted those that left the deepest marks, using them as a reference to understand and express the magnitude of a child’s loss. Grief is described by Freud^(20)^ as a process of loss of interest in things and a great and painful dejection, which relates to the situation of comparison of griefs addressed by interviewees, in which all are similar and can be equally painful.

Each individual is unique, navigating life with their own singularity. Similarly, the grieving process is a personal experience that triggers distinct responses and reactions in each person. In the literature, it is possible to find diverse approaches to understanding this human phenomenon in reaction to loss, but the basis for its comprehension lies in considering its multifaceted nature and in a comprehensive view of bereaved people in all aspects of life^(20,21)^.

Shock can arise in response to highly stressful situations, such as the news of a child’s death, which generally results in a state of emotional imbalance^(22,23)^. Bereaved individuals who have experienced shock from death are more vulnerable to mental health problems related to grief, as well as post-traumatic stress disorder^(22)^. Psychological trauma occurs within a specific context experienced by a person, from which they cannot escape and for which they lack sufficient resources to cope^(23)^. This demonstrates the importance of access to professional support and care, given that health impacts can accompany a person throughout their life^(23)^.

According to Kübler-Ross^(6)^, questions about the cause of death indicate the “denial” stage, as well as the non-acceptance of loss present in the DCSs. The sadness and the desire to no longer want to live evidenced in the DCSs are signs of the “depression” stage. Elisabeth Kübler-Ross^(6)^ argued that the five stages of grief are not linear and that some people may not experience all of them, although they always end in “acceptance”.

A child’s birth usually brings joy to families, but when loss occurs before the first moment of togetherness, as in the case of stillbirths, it is a challenging experience, resulting in suffering, pain, and a change from what was expected for life^(24)^. This experience, in situations of stillbirth, often triggers profound sadness, depression, fear, anger, and social isolation, and can characterize a complicated grief process^(24)^.

However, there is also the view that the lasting connection between parents and adult children can intensify grief when there is a loss^(25,26)^. Generally speaking, losing a child is a painful and challenging experience, and in both circumstances, there is a greater risk of grief complications, requiring professional intervention so that the bereaved can process it adequately^(25,26)^.

In relation to grief duration, the DCSs are divided, demonstrating situations with greater difficulty and suffering, and also the development of adaptive processes to cope with the loss, enabling life to continue with optimism and willpower. There is recognition that the absence of the loved one will be permanent, but that there are other important aspects of life that deserve attention and that can serve as support for a bereaved person.

There is no fixed time frame for grieving, as it is unique to each person, taking into account their individuality^(21)^. It is crucial to monitor the grieving process and the feelings associated with loss, which can persist throughout life without necessarily indicating a depressive disorder^(21)^. Indifference towards life and eating difficulties, for instance, can be signs of complicated grief, requiring assessment by a qualified professional, depending on its duration^(21)^.

For the older adults interviewed, wearing dark clothes and practicing closed grief can help express the pain of loss, communicating grief nonverbally to those around them. The reports indicated that visiting the cemetery can bring comfort and a sense of closeness to the loved one, as well as assisting in the grieving process, especially in what Freud^(20)^ called the Reality Testing, the moment when a bereaved person repeatedly confronts the reality of a loss.

Religiousness and spirituality can be important resources for successful aging, requiring daily practice^(27)^. They represent a profound and mature dimension of life in old age, offering support to face losses and challenges^(27)^. Research conducted in Kansas, United States, with older adults aged 65 or more, demonstrated that social support from friends and spiritual coping strategies were factors related to low levels of depressive symptoms in this group^(28)^. Religiosity and spirituality also frequently influence how people experience illness, death, and grief^(29)^. In the present study, it was evident in the DCSs that cultivating spirituality, such as through prayer, can help in the grieving process, alleviating anguish and promoting calm.

Positive thinking, in the context of resilience, can benefit health, as indicated by the results of this study. However, promoting it indiscriminately has the potential to hinder self-awareness of health problems, lead to denial of life’s challenges, and negatively impact quality of life^(27,30)^. Furthermore, in the dual process of grief^(31)^, there is an oscillation involving two states related to loss. In “loss stress”, a person focuses on emotions related to what was lost, seeking to understand reality through absence (looking at old photos, crying over the loss or imagining how they would react are some examples)^(31)^. In “restoration stress”, a bereaved person’s focus is on adapting without the loved one, seeking to do new things and reorganize their own life. Crocheting, visiting friends and preparing parties for children are strategies related to “restoration stress”^(31)^.

### Study limitations

A limitation of this study is that only Christian (Catholics and Lutherans) older adults participated. The use of the snowball sampling method did not allow for interviews with older adults who had other religions and who might have different worldviews and relationships with death. Since it is a subjective matter, each experience of grief is unique, which reinforces the need for further research that broadens the focus to other stages of life and to people with beliefs other than Christianity.

### Contributions to nursing

This study contributes to the field of nursing by highlighting an aspect of care that is still little discussed. Grief is an important determinant of health, well-being, and quality of life in the general population and, specifically in this study, among older adults. Nurses are in direct contact with people in their health care, so they can play an important role in supporting them through grief and providing comprehensive care. Thus, understanding the experiences of older adults regarding grief resulting from a child’s loss contributes to a closer connection between nursing professionals and issues in older adults’ lives that influence their health, at a time in life when deceased children could be fundamental sources of support.

## FINAL CONSIDERATIONS

Grief can be objectively defined as a concept; however, objectivity in defining it may disregard many unique aspects experienced by bereaved individuals. Definitions of grief are necessary to discuss the subject, but adhering to any one definition or basing oneself on a single theory can limit the interpretation of the phenomenon.

A child’s loss is a significant event that directly impacts older adults’ physical and mental health. Many aspects of life are affected by grief, which, when related to a child’s death, tends to be prolonged and involves the experience of intense feelings such as guilt, emptiness, longing, sadness, and even a loss of meaning in life. It has been found that although there are many losses throughout life, a child’s death is incomparable and difficult to accept.

Faith and positive thinking are coping strategies used by older adults. Furthermore, private grief and visits to the cemetery carry important symbolism in the grieving process. It is also essential to seek enjoyable activities, such as crocheting or interacting with friends, to help give new meaning to life after the loss.

In the face of a complicated bereavement situation - most common in parental bereavement - appropriate management by healthcare professionals is necessary. This includes acceptance, empathetic listening, and care, as well as the often-necessary shared care among the multidisciplinary team, thus prioritizing interdisciplinary and humanized practices.

## Data Availability

The research data are available only upon request.
