# Supplementary material for: Experiences of older adults who have lost a child: reflections on grief and for nursing
Source: Rev Bras Enferm. 2026 Jul 6;79:e20250260. doi: 10.1590/0034-7167-2025-0260 (PMC13336226; doi:10.1590/0034-7167-2025-0260)
Supplement: Translation

# Translation: Vivência de idosos que perderam um filho: reflexões sobre o luto e para a enfermagem

**DOI:** 10.1590/0034-7167-2025-0260pt

**Authors:** Richard Augusto Thomann Beckert, Valéria Silvana Faganello Madureira, Ivonete Teresinha Schulter Buss Heidemann, Tiago Luan Labres de Freitas, Angélica Zanettini Konrad, Samille Isabel Palombit Ronsoni, Maiara Bordignon

**Affiliations:** I Universidade Federal de Santa Catarina. Florianópolis, Santa Catarina, Brasil; II Universidade Federal da Fronteira Sul. Chapecó, Santa Catarina, Brasil

**Keywords:** Luto, Idoso, Saúde do Idoso, Relações Pais-Filho, Enfermagem.

## Abstract

**Objetivos:**

compreender a vivência do processo de luto em idosos que, em qualquer etapa da vida, perderam um filho.

**Métodos:**

pesquisa com abordagem qualitativa, da qual participaram oito idosos selecionados a partir do método de bola de neve. Foram realizadas entrevistas semiestruturadas, analisadas pela técnica do Discurso do Sujeito Coletivo.

**Resultados:**

as temáticas incluíram: Diferenças entre o luto por um filho e por outros familiares; Sentimentos provocados pela morte; Duração dos sentimentos e do luto; Sentimentos do luto que ficam; e Maneiras de lidar com a perda de um filho.

**Considerações Finais:**

embora a aceitação da perda seja possível, a saudade e a falta permanecem. Destacou-se a importância da fé e da rede de apoio para lidar com o luto. A enfermagem pode exercer papel importante de apoio no enfrentamento do luto. Compreender essas vivências é fundamental para a oferta de cuidados integrais.

## INTRODUÇÃO

A população brasileira está envelhecendo devido à redução da taxa de natalidade e ao aumento da expectativa de vida, causando uma inversão na pirâmide etária, fenômeno denominado “transição demográfica”^(1)^. Essa mudança resulta em crescente demanda por serviços de saúde e ferramentas para melhorar a qualidade de vida dos idosos, de forma que sejam ativos por mais tempo^(1)^.

O envelhecimento começa antes dos 60 anos, idade que define o indivíduo como pessoa idosa no Brasil^(2)^, caracterizando-se como um processo complexo de mudanças biopsicossociais que englobam transformações sociais, econômicas, físicas e psicológicas^(3)^. Assim, a senescência se refere ao envelhecimento natural, enquanto a senilidade é caracterizada por perdas mais acentuadas devido a complicações de saúde agudas ou crônicas^(3)^.

O envelhecimento pode ser visto como um processo de ganhos e perdas, em que o sucesso reside na capacidade de um indivíduo se adaptar às mudanças que o acompanham. As perdas durante a vida podem abranger vários aspectos, tais como diminuição do círculo social, capacidade funcional reduzida, mudança de papéis na sociedade e morte de conhecidos, amigos ou familiares^(4,5)^. O luto é uma resposta à perda, marcando o fim de um modo de relacionamento entre o indivíduo enlutado e o ente querido perdido, com mudanças na vida do enlutado^(4,5)^.

Compreender o luto pode ser difícil justamente por ser uma experiência subjetiva e peculiar àquele que o vivencia. Todavia, a psiquiatra Elisabeth Kübler-Ross, enquanto acompanhava pacientes terminais, desenvolveu uma teoria já bastante difundida, identificando cinco estágios do processo de luto: negação e isolamento; raiva; barganha; depressão; e aceitação^(6)^.

De acordo com tais estágios^(6)^, no estágio “negação e isolamento”, o indivíduo nega a perda como uma defesa inicial e temporária; no estágio “raiva”, o sentimento é de grande revolta e indignação diante da sensação de injustiça da perda; o estágio “barganha” diz respeito ao momento em que os sentimentos do enlutado estão amenizados e há a tentativa de negociar a perda e reverter a situação; no estágio “depressão”, o enlutado constata que não há como recuperar o que foi perdido e a melancolia predomina; e na “aceitação”, o enlutado retoma progressivamente a funcionalidade em sua vida. Embora nem todos os enlutados passem por todos os estágios, que são cíclicos e podem se alternar ao longo do processo de enfrentamento^(7)^, fica evidente que o luto pode trazer sentimentos e sensações diversas, que têm o potencial de influenciar significativamente a saúde de quem o vivencia.

Além disso, o ser humano se desenvolve em um ambiente social, e as relações entre pais e filhos geralmente se destacam pelo afeto e pela intimidade^(8)^. Assim, a perda de um filho pode ser um processo doloroso em qualquer fase da vida, com um luto mais intenso e prolongado em comparação a outras perdas^(8)^. À medida que envelhecem, muitas pessoas enfrentam a perda de um filho e a vivenciam de maneira única, o que tem impacto na saúde e pode requerer suporte de profissionais de saúde^(9)^. Muitas doenças têm início em situações difíceis pelas quais as pessoas passam, o que inclui os processos de luto^(10)^. O apoio profissional nesses casos, marcado por escuta e diálogo, é importante para amparar as pessoas no enfrentamento dessas situações^(10)^.

O profissional enfermeiro está na linha de frente dos serviços de saúde, o que o coloca em contato direto com pacientes, familiares e acompanhantes. Considerando-se o cuidado humano como foco da sua profissão, sensibilizar-se sobre questões como o luto é essencial para uma visualização ampliada do processo de saúde-doença, para garantia da integralidade e humanização do cuidado^(11)^. Nesse cenário, o papel do enfermeiro no cuidado à pessoa em luto envolve uma variedade de competências, que incluem tecnologias leves de cuidado como escuta, acolhimento e comunicação, além da implementação de intervenções e programas de acompanhamento ao enlutado, em atenção às suas necessidades culturais e espirituais, entre outros^(12)^.

Esse contexto demonstra a importância de se desenvolver, durante a formação de enfermeiros e após, quando inseridos nos serviços de saúde, conhecimentos e habilidades para acolhimento e apoio a pessoas e famílias enlutadas, garantindo a atenção às particularidades de cada situação^(12)^. Diante disso, emergiu a seguinte pergunta de pesquisa: como foi ou está sendo a vivência do luto por idosos que, em algum momento da vida, perderam um filho?

Para mapear a discussão da temática em nível nacional, realizou-se uma breve pesquisa no Banco de Teses e Dissertações da Coordenação de Aperfeiçoamento de Pessoal de Nível Superior com os termos “luto”, “idosos” e “filhos”. Foram encontrados nove resultados publicados entre 1997 e 2012, dos quais sete são dissertações de mestrado acadêmico, um, mestrado profissional, e um, tese de doutorado. Modificando a pesquisa para os termos “luto”, “idosos” e “enfermagem”, encontram-se também nove trabalhos desenvolvidos entre 2004 e 2009, sendo seis dissertações de mestrado e três teses de doutorado. Assim, diante da carência de pesquisas, nota-se quanto espaço ainda há para discussão acadêmica do luto em idosos que perderam um filho, seja na saúde, de modo geral, ou na enfermagem.

## OBJETIVOS

Compreender a vivência do processo de luto de idosos que, em qualquer etapa da vida, perderam um filho.

## MÉTODOS

### Aspectos éticos

A pesquisa foi aprovada pelo Comitê de Ética em Pesquisa da universidade de vínculo dos autores. Seguiu as normativas éticas para pesquisa com seres humanos, e assegurou o anonimato dos participantes por meio do uso da letra “E”, de entrevistado, seguida de número indicativo da ordem das entrevistas (E1; E2; E3; E4; E5; E6; E7; e E8).

### Tipo de estudo

Trata-se de estudo com abordagem qualitativa. Esse tipo de desenho é constituído por uma série de diferentes técnicas interpretativas com o objetivo de descrever e decodificar os componentes de um complexo sistema de significados^(13)^. Este relatório de estudo também considerou o guia *Consolidated criteria for Reporting Qualitative research* ^(14)^.

### Cenário do estudo

O estudo contou com a participação de idosos de um município localizado no oeste do estado de Santa Catarina, Brasil, que vivenciaram o luto pela morte de um filho.

### Fonte de dados

Oito idosos que vivenciaram o luto pela morte de um filho participaram do estudo. O número de participantes foi determinado por saturação de dados^(15)^, indicando o fim da coleta quando as respostas começaram a se repetir e o sentido do fenômeno/grupo estudado foi compreendido. Os participantes foram selecionados usando o método de bola de neve^(16)^ (do inglês, *snowball sampling*), uma amostragem baseada em referências sucessivas.

Inicialmente, dois idosos, conhecidos da pesquisadora responsável a partir de suas atividades teórico-práticas desenvolvidas em uma Unidade Básica de Saúde, foram convidados por meio de aplicativo de mensagens. Posteriormente, esses idosos indicaram outros que vivenciaram a perda de um filho a serem convidados, continuando o processo de convite e inclusão no estudo até alcançar o número de oito participantes. Foram incluídas no estudo pessoas com mais de 60 anos e que tinham vivenciado o luto por um filho durante a sua vida. Foram excluídos idosos que não residiam no município estudado. Salienta-se que foi estabelecido contato telefônico com todos os participantes antes da entrevista, para apresentação do estudo, do entrevistador e para obtenção do aceite à participação, com posterior assinatura do termo de consentimento.

### Coleta e organização dos dados

A coleta de dados foi norteada por um roteiro de questões desenvolvido para este estudo. As entrevistas foram realizadas por dois dos autores nas casas dos participantes entre julho e outubro de 2023. Os participantes receberam orientações quanto à gravação das entrevistas e concordaram com ela. Os entrevistadores eram estudantes de um curso de graduação em enfermagem, sendo um do sexo masculino e outro do sexo feminino, que atuaram sob orientação direta de uma docente doutora em enfermagem. As transcrições foram feitas imediatamente após cada entrevista. Ressalta-se que, durante a coleta de dados, foram contactados nove idoso, porém um deles manifestou sua recusa após duas tentativas de entrevistá-lo sem sucesso.

### Análise dos dados

Os dados obtidos foram transcritos e analisados de acordo com o Discurso do Sujeito Coletivo (DSC)^(17)^, técnica que analisa os depoimentos e identifica expressões-chave, ideias centrais (ICs) e ancoragens, para compreender as manifestações linguísticas de pensamentos sobre uma ideia central. Inicialmente, realizou-se uma leitura minuciosa das respostas de cada participante para identificar as expressões-chave, que representavam a essência do depoimento. Em seguida, foram identificadas as ICs, que são expressões sintéticas que transmitem o sentido de cada discurso. Por fim, as expressões-chave que compartilhavam a mesma ideia central foram agrupadas para formar o DSC^(17)^. Das análises das entrevistas, emergiram nove temas, 25 ICs e 31 DSCs, cinco dos quais, juntamente com suas respectivas ICs e DSC, foram explorados neste estudo.

## RESULTADOS

Quanto ao perfil dos oito idosos, elaborou-se o Quadro 1 que apresenta idade, sexo, ocupação, situação conjugal, número de filhos, número de netos, religião e número de filhos falecidos.

**Quadro 1:** Dados demográficos dos participantes

	Idade	Sexo	Ocupação	Situação conjugal	Nº de filhos	Nº de netos	Religião	Filhos falecidos
**E1**	66	F	Aposentada	Divorciada	2	1	Católica	1
**E2**	77	F	Aposentada	Viúva	8	22	Católica	1
**E3**	95	F	Aposentada	Viúva	12	22	Católica	3
**E4**	63	F	Aposentada	Divorciada	2	0	Luterana	2
**E5**	65	F	Aposentada	Viúva e divorciada	14	9	Católica	6
**E6**	74	F	Cuidadora de crianças	Divorciada	6	5	Católica	3
**E7**	62	M	Pedreiro	Viúvo	2	0	Católica	1
**E8**	69	F	Aposentada	Viúva	4	5	Católica	1

A seguir, estão apresentadas cinco temáticas que emergiram da análise, com suas respectivas ICs e DSCs.

### (1) Diferenças entre o luto por um filho e por outros familiares

IC - Foram muitas perdas, mas perder um filho é incomparável.

DSC - Foram muitas perdas e foram pesadas. Tive muitos velórios de parentes, mas, por exemplo, na morte dos pais, o luto é mais leve porque você pensa que eles tinham que ir, como eu terei que ir um dia. Quando morre esposo ou esposa, o luto é mais passageiro. Mas a vivência de ver seu filho chegar em casa, te dar um abraço, um beijo, pedir uma benção, isso que é diferença! Perder um filho é incomparável! Quando se perde um filho, parece que um pedaço de você morreu. A dor é muito mais do coração mesmo. O sofrimento é bem maior do que em outros lutos, pois parece que o coração está despedaçado, que tem uma ferida.

IC - Todas as perdas são parecidas.

DSC - Eu acho que é tudo parecido. Por exemplo, os filhos são do mesmo sangue do marido. Então, não tem como esquecer. A gente só esquece depois que morre, né?

### (2) Sentimentos provocados pela morte

IC - No início, levei um choque e achei que fosse brincadeira.

DSC - No primeiro momento, levei um choque tão grande que não senti mais nada e achei que fosse uma brincadeira. Depois, veio um desespero, fiquei sem acreditar no que estava acontecendo e quase desmaiei, me senti arrasada. Senti muita dor no coração por não poder fazer nada e fiquei questionando o porquê do ocorrido, sem resposta.

IC - Naquele momento, meu mundo acabou e ficou só a tristeza.

DSC - A minha vida parou ali e não tinha mais sentimento nenhum dentro, não pensava mais no que tinha que fazer, não pensava em mais nada, pois o meu mundo fechou e ficou a tristeza, muita tristeza. Sinto que ali o meu mundo acabou. Fiquei sem chão e não queria mais viver. É uma situação muito triste, meu filho era meu xodó!

IC - Perder um filho é ainda mais triste quando você conviveu com ele.

DSC - Meu sonho era ter gêmeos! Eu tive a graça e não pude conviver com eles. Então, se perder um recém-nascido já é triste, imagina um filho com quem você conviveu!

### (3) Duração dos sentimentos e do luto

IC - O luto dura muito tempo.

DSC - O luto dura muito tempo. Antes eu me desesperava! Você pode perder tudo, mas um filho é muito difícil! Eu continuo com aquele aperto no coração. Sinto que não sou mais aquela pessoa, que não tenho mais aquela alegria que tinha.

IC - O luto e a falta não passam.

DSC 1 - Ainda hoje é luto. Não passou e não vai passar. Sinto falta, sinto saudades deles, e essa não vai passar. Até hoje uso preto direto e tomo remédio para depressão. Minha vida acabou. Então, não quero fazer mal para ninguém, mas eu quero morrer.
DSC 2 - Eu aprendi a viver com isso, mas a falta dele vai ser difícil passar. Sei que a vida continua, que não é porque meu filho faleceu que eu faleci junto, né? Tenho outros filhos, e por isso tenho que manter a força para superar e dar suporte para os que ficaram.

### (4) Sentimentos do luto que ficam

IC - É algo que se lembra todos os dias e se sente saudades.

*DSC - Eu lembro todos os dias. Não tem um momento em que esqueço daquilo! Eu tento superar ao máximo, mas está sempre presente na minha vida. Sempre tem aquela dorzinha e ela pesa. Sinto uma dor cansada e pesada, que alivia só quando eu choro. Isso me machuca bastante. Então, hoje o sentimento é de saudade, sinto muito a falta dele. Saudade de um abraço e dos momentos que vivemos juntos.*

IC - Não consigo aceitar a perda.

DSC 1 - Eu gostaria que ele estivesse com a gente. Não consigo entender! Não aceito a perda. Às vezes, penso que vou morrer e não vou ficar sem ele.
*DSC 2 - Às vezes, acho que está viajando e vai voltar, parece que está voltando para casa, mas sei que não está. Tento superar, mas dizer que ele não existiu, não dá! Não sei explicar o que acontece. Hoje não tenho mais vontade de fazer nada, de trabalhar, mas não vou ficar parado em casa. Perdi o ânimo.*

### (5) Maneiras de lidar com a perda de um filho

IC - A fé, a oração e o pensamento positivo ajudaram muito.

DSC - Em primeiro lugar, muita fé em Deus! A oração ajuda muito, por isso procurei muito a igreja, e isso me ajudou. Eu sou muito de rezar, e um dia resolvi fazer uma novena à Nossa Senhora do Caravaggio. Isso foi me aquietando o coração e comecei a dormir bem, a chorar menos. Então, foi a fé e pensar sempre positivo.

IC - Usar luto fechado me ajudou.

DSC - Antigamente, era luto fechado mesmo, usando roupas pretas de seis meses até um ano! Fazer diferente era contra Deus. Eu acho que isso ajudou, pois eu precisava passar aquele tempo, por aquela situação.

IC - Ir ao cemitério me conforta.

DSC - Tem dias que vou no cemitério. Fico o dia inteiro lá e não sinto vontade de sair porque o jazigo que fiz para ele me aquieta o coração. Ir lá limpar o jazigo, conversar e cuidar dele. Coloquei coroa de flores, fotos e sigo visitando o cemitério para rezar. Isso me conforta.

IC - Eu faço muitas coisas para me ajudar.

*DSC 1 - Procuro fazer crochê, visitar uma amiga para me aquietar e não ter o pensamento só naquilo* [na morte do filho]. *Além disso, busco trabalhar, ir fazendo as coisas e seguindo a vida como eu posso. Eu tento, ao máximo, ser forte, pois, se eu não me ajudasse e tivesse força, não conseguiria superar, né?*
DSC 2 - Depois que perdi meu filho recém-nascido, faço uma festinha para 12 crianças no Dia das Crianças. Eu dou presente e faço o possível para agradá-las. Faço isso para tentar preencher aquele vazio que nunca preenche.

Sequencialmente, a Figura 1 representa graficamente os impactos da perda de um filho na vida dos idosos participantes. Nela, uma linha pontilhada horizontal simboliza a morte do filho, enquanto duas setas espirais representam o processo de luto. A espiral ascendente, colorida em verde, representa um luto elaborado sem complicações, enquanto a espiral descendente, em vermelho, representa um luto complicado. Uma linha vertical corta as espirais, simbolizando a falta permanentemente sentida pelos idosos após a perda. As palavras nas espirais representam elementos associados à experiência do luto, conforme descrito nos resultados.

**Figura 1:** Representação gráfica do luto vivenciado pela perda de um filho, Chapecó, Santa Catarina, Brasil

## DISCUSSÃO

Para alguém que já passou dos 60 anos, a perda de familiares e a vivência do luto são algo em geral já conhecido^(18)^. O impacto que a falta causará na vida do enlutado está relacionado ao vínculo e ao afeto que havia entre o ente querido e a pessoa que ficou. O vínculo entre pais e filhos frequentemente é forte em afeto, de tal forma que o rompimento dele provoca grande sofrimento e pode exigir maior esforço para adaptar-se à nova realidade e para elaborar o luto^(9,19)^.

Durante as entrevistas, ao serem questionados sobre outras experiências de perda significativa em suas vidas, os idosos destacaram aquelas que deixaram marcas mais profundas, usando-as como referência para compreender e expressar a magnitude da perda de um filho. O luto é descrito por Freud^(20)^ como um processo de perda de interesse pelas coisas e um grande e doloroso abatimento, o que remete à situação de comparação dos lutos abordada pelos entrevistados, em que todos são parecidos e podem ser igualmente dolorosos.

Cada indivíduo é único, navegando pela vida com sua própria singularidade. Da mesma forma, o processo de luto é uma vivência pessoal que desencadeia respostas e reações distintas em cada pessoa. Na literatura, é possível encontrar diversas abordagens para compreender esse fenômeno humano em relação à perda, mas a base para sua compreensão reside na consideração de sua multifacetada natureza e na visão abrangente do enlutado em todos os aspectos da vida^(20,21)^.

O choque pode surgir em resposta a situações de grande estresse, como a notícia da morte de um filho, o que geralmente resulta em um estado de desequilíbrio emocional^(22,23)^. Pessoas enlutadas que passaram por choque com a morte estão mais vulneráveis a problemas de saúde mental e relacionados ao luto, assim como transtorno de estresse pós-traumático^(22)^. O trauma psicológico ocorre diante de um contexto específico vivenciado por uma pessoa, do qual não consegue escapar e não possui recursos suficientes para o seu enfrentamento^(23)^. Isso demonstra a importância do acesso ao acolhimento e apoio profissional, tendo em vista que os impactos à saúde podem acompanhar a pessoa por toda a vida^(23)^.

À luz de Kübler-Ross^(6)^, as perguntas sobre o motivo da morte indicam o estágio de “negação”, bem como a não aceitação da perda presente nos DSCs. A tristeza e o desejo de não querer mais viver evidenciados nos DSCs são sinais do estágio de “depressão”. Elisabeth Kübler-Ross^(6)^ defendia que os cinco estágios do luto não são lineares e que algumas pessoas podem não vivenciar todos eles, embora sempre terminam na “aceitação”.

O nascimento de uma criança costuma trazer alegria às famílias, mas quando ocorre a perda antes do convívio, como no caso dos natimortos, é uma experiência desafiadora, pois resulta em sofrimento, dor e mudança do que era esperado para a vida^(24)^. Esta vivência, em situações de natimortalidade, frequentemente desencadeia tristeza profunda, depressão, medo, raiva e isolamento social, podendo caracterizar um luto complicado^(24)^.

Porém, existe também a visão de que a conexão duradoura entre pais e filhos adultos pode intensificar o sofrimento quando há uma perda^(25,26)^. De maneira geral, perder um filho é uma experiência dolorosa e desafiadora, e, em ambas as circunstâncias, há um maior risco de complicação do luto, exigindo intervenção profissional para que o enlutado possa elaborá-lo adequadamente^(25,26)^.

Em relação ao tempo de duração do luto, os DSCs se dividem, demonstrando situações com maior dificuldade e sofrimento, e também o desenvolvimento de processos adaptativos para lidar com a perda, possibilitando que a vida siga com otimismo e força de vontade. Há o reconhecimento de que a ausência do ente querido será permanente, mas que há outros aspectos importantes na vida que merecem atenção e que podem servir de apoio para a pessoa enlutada.

Não existe um tempo fixo para viver o luto, pois é único para cada pessoa, levando em conta sua individualidade^(21)^. É crucial acompanhar o processo de luto e os sentimentos associados à perda, que podem persistir ao longo da vida sem necessariamente indicar um transtorno depressivo^(21)^. Indiferença perante a vida e dificuldades alimentares, por exemplo, podem ser sinais de luto complicado, exigindo avaliação por profissional qualificado, dependendo da sua duração^(21)^.

Para os idosos entrevistados, o uso de roupas escuras e a prática de um luto fechado podem ajudar a expressar a dor da perda, comunicando de forma não verbal o pesar às pessoas ao seu redor. Os relatos indicaram que visitar o cemitério pode trazer conforto e a sensação de proximidade com o ente querido, bem como auxiliar na elaboração do luto, em especial no que Freud^(20)^ chamou de Teste de Realidade, momento em que o enlutado confronta repetidamente a realidade da perda.

A religiosidade e espiritualidade podem ser recursos importantes para um envelhecimento bem-sucedido, requerendo prática diária^(27)^. Elas representam uma dimensão profunda e madura da vida na terceira idade, oferecendo apoio para enfrentar perdas e desafios^(27)^. Em pesquisa realizada em Kansas, Estados Unidos, com idosos com 65 anos ou mais, demonstrou-se que o apoio social de amigos e estratégias de enfrentamento espiritual foram fatores que se relacionaram ao baixo nível de sintomas depressivos neste grupo^(28)^. A religiosidade e espiritualidade também influenciam com frequência a forma como as pessoas vivenciam suas experiências de doença, morte e luto^(29)^. No presente estudo, ficou evidenciado nos DSCs que cultivar a espiritualidade, tal como através da oração, pode ajudar no processo de luto, aliviando a angústia e promovendo calma.

O pensamento positivo, no contexto da resiliência, pode beneficiar a saúde, conforme indicaram os resultados deste estudo. Contudo, promovê-lo indiscriminadamente tem o potencial de impedir a autopercepção de problemas de saúde, levar à negação dos desafios da vida e impactar negativamente a qualidade de vida^(27,30)^. Ademais, no processo dual do luto^(31)^, há uma oscilação envolvendo dois estados que se relacionam à perda. No “estresse de perda”, a pessoa se concentra nas emoções relacionadas ao que foi perdido, buscando compreender a realidade através da ausência (olhar fotos antigas, chorar pela perda ou imaginar como ele ou ela reagiria são alguns exemplos)^(31)^. No “estresse de restauração”, o foco do enlutado é adaptar-se, sem o ente querido, buscando fazer coisas novas e reorganizar a própria vida. Fazer crochê, visitar amigos e preparar festas para crianças são estratégias relacionadas com o “estresse de restauração”^(31)^.

### Limitações do estudo

Como limitação do estudo, considera-se o fato de que somente idosos cristãos (católicos e luteranos) participaram. O uso do método bola de neve não possibilitou entrevistas com idosos que tinham outras religiões e que poderiam ter outras cosmovisões e relações com a morte. Por ser um assunto subjetivo, cada vivência do luto é única, o que reforça a necessidade de novas pesquisas que ampliem o foco para outras etapas da vida e para pessoas com outras crenças que não o cristianismo.

### Contribuições para a área da enfermagem

O estudo contribui para a área da enfermagem ao evidenciar um aspecto do cuidado ainda pouco discutido. O luto é um importante fator determinante da saúde, bem-estar e qualidade de vida na população geral e, especificamente, neste estudo, entre idosos. O enfermeiro está em contato direto com as pessoas no cuidado da saúde delas, de modo que pode exercer papel importante de apoio no enfrentamento do luto e na oferta de cuidados integrais. Desse modo, a compreensão das vivências de idosos com relação ao luto decorrente da perda de um filho contribui para maior aproximação dos profissionais de enfermagem com questões da vida dos idosos e que influenciam a saúde, em um momento da vida no qual os filhos que faleceram poderiam ser fontes fundamentais de apoio.

## CONSIDERAÇÕES FINAIS

O luto pode ser definido objetivamente como conceito; entretanto, a objetividade em defini-lo pode desconsiderar muitos aspectos peculiares vivenciados pelas pessoas enlutadas. As definições do luto são necessárias para discutir o assunto, mas prender-se a qualquer uma delas ou fundamentar-se em uma única teoria pode limitar a interpretação do fenômeno.

A perda de um filho é uma situação relevante e que influencia diretamente a saúde física e mental dos idosos. Muitos aspectos da vida são afetados pelo luto, que, quando relacionado à morte de um filho, tende a ser prolongado e envolve a experiência de sentimentos intensos como culpa, vazio, saudade, tristeza e até mesmo a perda do sentido da vida. Constatou-se que embora existam muitas perdas ao longo da vida, a morte de um filho é incomparável e difícil de ser aceita.

A fé e o pensamento positivo são estratégias de enfrentamento utilizadas pelos idosos. Além disso, o luto fechado e as visitas ao cemitério carregam um simbolismo importante para elaboração do luto. Também é essencial buscar atividades prazerosas, como o crochê ou a interação com amigos, para auxiliar a ressignificar a vida após a perda.

Diante de uma situação de luto complicado - mais comum em luto parental -, é necessário o manejo adequado pelos profissionais de saúde. Isso inclui acolhimento, escuta empática e cuidado, bem como a necessidade muitas vezes de um cuidado compartilhado entre a equipe multiprofissional, priorizando, assim, as práticas interdisciplinares e humanizadas.

## Data Availability

Os dados de pesquisa só estão disponíveis mediante solicitação.
